# Synesthesia and music perception

**DOI:** 10.1590/S1980-57642015DN91000004

**Published:** 2015

**Authors:** Guilherme Francisco F. Bragança, João Gabriel Marques Fonseca, Paulo Caramelli

**Affiliations:** 1 PhD Student, Neuroscience Program, The Federal University of Minas Gerais, Belo Horizonte, MG, Brazil.; 2 MD,PhD, Department of Internal Medicine, Faculty of Medicine, and Department of General Theory of Music, Music School, The Federal University of Minas Gerais, Belo Horizonte, MG, Brazil.; 3 MD, PhD, Neuroscience Program, The Federal University of Minas Gerais, Belo Horizonte, MG, Brazil; and MD, PhD, Department of Internal Medicine, Faculty of Medicine, The Federal University of Minas Gerais, Belo Horizonte, MG, Brazil

**Keywords:** synesthesia, cross-modal association, perception, music

## Abstract

The present review examined the cross-modal association of sensations and their relationship to musical perception. Initially, the study focuses on synesthesia, its definition, incidence, forms, and genetic and developmental factors. The theories of the neural basis of synesthesia were also addressed by comparing theories emphasizing the anatomical aspect against others reinforcing the importance of physiological processes. Secondly, cross-modal sensory associations, their role in perception, and relationship to synesthesia were analyzed. We propose the existence of a lower, unconscious degree of synesthesia in non-synesthetes. This latent synesthesia (without explicit sensory manifestations) would be functional, aiding the construction of abstract associations between different perceptual fields. Musical meaning might be constructed largely by synesthetic processes, where the sensory associations from sound activate memories, images, and emotions.

## Abstract (pt)

No presente trabalho de revisão, investigamos o cruzamento de sensações e sua relação com a percepção musical. Inicialmente, nos debruçamos sobre o estudos da sinestesia, sua definição, incidência, formas, fatores genéticos e de desenvolvimento. Abordamos ainda as teorias sobre as bases neurais da sinestesia, comparando teorias que enfatizam os aspectos anatômicos a outras que reforçam a importância de processos fisiológicos. Numa segunda seção tratamos dos cruzamentos de modos sensoriais, seu papel na percepção e sua relação com a sinestesia. Cogitamos haver um grau de sinestesia mais baixo e inconsciente em não sinestetas, esta sinestesia latente (sem manifestações sensoriais explícitas) seria funcional, auxiliando na construção de associações abstratas entre campos perceptivos diferentes. A significação musical talvez seja construída, em grande parte, por processos sinestésicos, em que as associações sensoriais a partir do sonoro ativam memórias, imagens e emoções.

## INTRODUCTION

The use of extramusical adjectives to describe the expressive character of a piece of music (heavy, light, brilliant, sweet, and dark, among others) or the syntactic characteristics of its composition (density, texture, straightness, and verticality, among others) is quite common, especially in the context of classical music. Although these terms usually qualify sensations other than sound, their use seems inevitable when discussing music. Such linguistic mixing is termed synesthesia, a figure of speech consisting of translating meanings, attributed to a perceived sensory impression typical of other sensations. Thus, a piece of music (sound event) could be considered sweet (sense of taste), rough (tactile) or brilliant (visual). Non-sonorous sensory terms and emotional qualifiers are adjectives often found in musical scores. Many of the indications of progress are kinetic (motion) adjectives, including "andante", "lento", and "presto", or emotional adjectives, including "adagio" (adagiare means accommodating carefully), "sostenuto" (steady, sustained), "commodo", "allegro", and "vivace".

Resorting to other sensations is necessary to discuss sound/music, suggesting that music perception is only achieved through interaction with other sensory fields. The much-repeated use of this linguistic resource may not be just a speech peculiarity; but rather may also provide clues about the mechanisms of information integration needed for the construction of perception.

**Synesthesia.** Synesthesia is also studied by neuroscientists, with the more restricted meaning of a neurological condition in which a stimulus (termed an inductor), which may be sensory (especially a sound or flavor) or cognitive (including a word, a number, or the names of days or months), involuntary, automatically, and consistently arouses a non-externally stimulated sensation (termed a concurrent). The defining characteristics of synesthesia as a neurological condition are outlined in more detail below:^1^

- To be involuntary and automatic means that synesthesia is not a conscious decision or a rational manifestation. Synesthesia is a passive, non- suppressible experience, although is aroused by an easily identifiable stimulus.
- To be consistent means that sensations evoked by stimuli do not change over time. Re-tests over a one-year period have shown consistency of over 90% in synesthesia in which the competing sensation was color viewing, according to Hubbard and Ramachandran.^2^
- It is idiosyncratic - synesthesia manifests itself in a personal way for the same stimulus. Thus, in grapheme-color synesthesia, each individual perceives the same letter as having a specific color.
- Most types of synesthesia are unidirectional, a number may evoke a color, although the color will not evoke the same number. However, cases of bi-directional synesthesia have been reported.^3^
- Synesthesia is additive; that is, it adds to the normal perception and does not replace or mask it.
- Synesthesia is an emotional experience; the synesthete has the conviction that that perception is significant and real. Many synesthetes feel shocked when they discover that other people do not share the same form of perception.^4^

The estimated prevalence of synesthesia in the population has varied greatly as the study of the subject has progressed. Initial studies indicated a rate of 1 in 25,000.^1^ In 2006, Simner and colleagues^5^ tested two large samples, one comprising 500 and another of 1190 subjects, and diversified the tests to measure different variants of synesthesia. The authors identified a prevalence of 1 in 23, evenly distributed between women and men. However, this estimated prevalence remains imprecise because not all possible variants of synesthesia are known. Sean Day has catalogued more than 65 forms of synesthesia on his website.^6^ Cytowic and Eagleman^7^ however, estimate the existence of more than 150 different forms of this condition.

**Figure 1:**
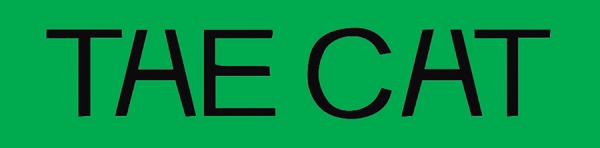
The two words may be easily read as "the cat", although the H and A have been written exactly the same way.^47^

Synesthesia apparently has a strong cognitive component because most grapheme-color synesthetes tend to associate the same color with a letter, regardless of its format in uppercase or lowercase, italic, bold, or different font faces (albeit sometimes with varying shades). Similarly, mixed signals (including the character l) tend to have different associations, depending on whether they are construed as letters (as the letter l in the word "land", for example) or as numbers (as the number 1). Even identical signals may be experienced with different colors in different contexts, including in the spelling of a word, as in Figure 1. The grapheme-color synesthete usually experiences the two ambiguous letters in Figure 1 as different colors because of the context.^7^ These findings indicate that synesthesia is likely aroused by cognitive processes of the highest level, including linguistic categories, and not (merely) by visual or acoustic sensations.^8^

Simner^8^ also argues that consistency, which has been considered the "gold standard" for the identification of synesthesia,^9^ is an essential feature of the synesthesia condition. In typical consistency tests, probable synesthetes undergo a surprise retest approximately six months after the first, which needs to have more than an 80% match with the previous test to confirm the neurological condition. The control group undergoes a retest approximately two weeks after the first with approximately 20% matching responses. However, there are groups with intermediate scores and others who claim to have sensations/cognitions automatically aroused by some stimulus, although the concurrent manifestation varies over time. Therefore, Simner raised the hypothesis that consistency forms a subgroup of synesthetes, although is not an essential feature of the condition.

Idiosyncrasy apparently is not an essential condition of synesthesia either. There is a strong association between treble and brighter colors, bass and darker shades, loud sounds and large shapes, and soothing sounds and small shapes in synesthesia where the inducing stimulus is sound.^10^ Music listening heavily relies on these sensory cross-modal associations that we automatically and involuntarily experience and which are largely shared.

Synesthesia as a neurological condition differs from adventitious synesthetic experiences resulting from the use of hallucinogenic drugs, epileptic seizures, visual disturbances, or tumors because it is innate, permanent, and has no effect on everyday life function. A study from the 1990s suggests that synesthesia mainly occurs in women, at a ratio of 3:1,^1^ although at least one subsequent study^5^ failed to corroborate this gender asymmetry.

Synesthesia was not included in the Fourth or Fifth Edition of the Diagnostic and Statistical Manual of Mental Disorders (DSM-IV),^6,11^ although adventitious synesthesia may appear as a symptom of psychiatric illness. Synesthesia seems to have genetic origins, given the incidence of synesthetes found in the same family. No case of father-to-son transmission of synesthesia had been reported by the beginning of the first decade of this century; only father-daughter, mother-son, and mother-daughter transmissions were reported, thereby indicating an X chromosome-linked transmission.^12-14^ However, a broader study (Asher et al., 2009), including 196 subjects from 43 families with cases of auditory-visual synesthesia, failed to confirm the linkage to chromosome X, although did indicate oligogenic transmission. In addition, two cases of father-to-son transmission were detected in the study.

Asher et al.^15^ collected DNA samples from each subject and analyzed approximately 410 microsatellites dispersed on chromosomes. Microsatellites are short sequences, repeated several times, in which each allele (variant) of a given locus contains a unique number of repeats, and this number varies between individuals. Thus, these sequences are used to identify the genetic variation in humans, that is, how different alleles of the same locus differ among individuals. To seek evidence of genetic linkage, the researchers compared the DNA samples of different generations of synesthetes from the same family to identify inherited microsatellites. They identified four different chromosome regions, located on three different chromosomes, where genes would be likely linked to synesthesia, regions also known to contain genes associated with a variety of disorders, including autism, dyslexia, and epilepsy.

The strongest linkage identified in the study was in a gene involved in the regulation of reelin, a key protein in the control of neuronal migration processes in the developing brain. Reelin maintains its activity in the adult brain, modulating synaptic plasticity and improving the induction and maintenance of long-term potentiation (LTP).^16^ The results of Asher et al.^15^ suggest that the genetic basis for synesthesia must lie at least partly in genes that affect the development of brain connectivity.

The tendency to develop synesthesia is apparently hereditary because research studies have reported cases of co-occurrence of rather different synesthesias in the same subject or in the same family,^17-19^ although the specific type of synesthesia might be determined by other factors.^19^ Rouw and Scholte^20^ proposed that newly learned categories tend to associate with categories assimilated earlier in children with a predisposition for synesthesia, causing the newer material to become the inductor and the older a competitor. This would explain why colors are usually competitors for graphemes, yet rarely the other way around. However, this hypothesis has key exceptions: sound sensations, usually categorized very early, are almost invariably inducers and rarely competitors.^21-23^

**Figure 2:**
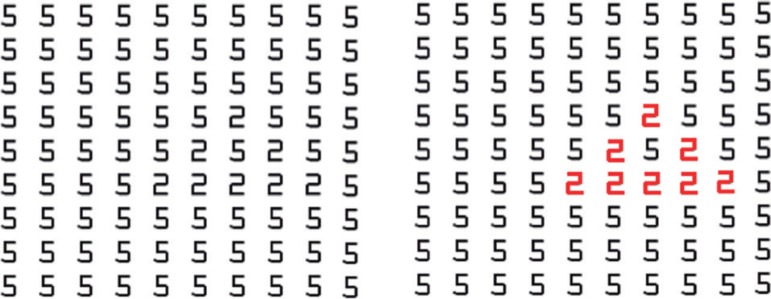
The synesthete detects the "2" numerals with same speed in both frames.^43^

In any case, many studies strongly indicate that synesthetic associations are developed through interactions with the environment.^3,19,24^ The most frequent synesthesia is the association of graphemes with colors, with a prevalence of approximately 1.4% in the general population, according to Day.^6^ An effective test to prove this type of synesthesia is depicted in the Figure 2, which shows all numerals written in gray in the first frame (either letters or other types of graphemes). The grapheme-color-type synesthete identifies the "2" numerals in the all-gray frame with the same speed and accuracy as a person without this synesthetic condition would in the second frame, where the "2" numerals are written in a different color, because the synesthete sees each character in a different color even when they are really all the same color (Figure 2).

Another synesthesia whose prevalence is as great as, or greater than, grapheme-color-type synesthesia is mirror-touch synesthesia. The act of observing another person being touched induces tactile sensations in their own body in this form of synesthesia.^25^ Researchers estimate that the prevalence of this synesthesia in the population is 1.6%. In a task used to confirm cases of mirror-touch synesthesia, the subject is touched in the cheeks or hands without seeing the object that touches them, while observing a person or object being touched. The subject is asked to mention the place where they were touched and to ignore the touches observed. Unlike the control group, synesthetes tend to be significantly faster in reporting touches when they coincide with those observed than when the actual and observed touches occur in different places. They also commit more errors than the control group, confusing the real touches with the observed ones.

Some hypotheses regarding the neural basis of this phenomenon are being explored, most raised by forms of synesthesia wherein an auditory, visual, or tactile stimulus arouses experiences of seeing colors.^4^ There are two parallel discussions regarding the neural basis of synesthesia, according to Hubbard and Ramachandran.^2^ The first is at the anatomical and physiological level and is centered on the discussion that synesthesia may occur through synaptic pruning failure or a disinhibition process. Synaptic pruning failure, an explanation of a more structural nature, is based on the theory that we have many connections at birth and that those connections are reduced throughout childhood, leaving only the necessary and most efficient functional connections.

Spector and Maurer,^24^ in a review article, noted that there is indirect evidence of an overabundance of connections between sensory cortical areas in early childhood and that pruning is dependent on experience. The authors cite various studies to exemplify this overabundance of connections. In one of the studies,^26^ tactile stimulation of the wrist of adults and children evokes normal activity in the somatosensory cortex, although this activation is only increased in newborns when the touch is accompanied by a sound stimulus of white noise, which is an indeterminate sound formed by the combination of all audible frequencies. In another example,^27^ spoken language triggers not only the activation of the auditory cortex in babies but also the visual cortex, and this activation does not cease until three years of age. Spector and Maurer^24^ cite as an example of experience-dependent pruning the fact that in adults who were blind since childhood, reading Braille or embossed Roman letters or performing tactile tasks recruits the visual cortex, including both the extrastriate cortex and the primary visual area.^28^

The alternative explanation of synesthesia is based on the process of disinhibited feedback from higher cortical areas onto sensory cortical areas: the primate cortex is arranged in parallel hierarchical structures, that is, simpler processing structures send signals to more complex structures.^29^ Thus, primary processing structures send signals to more complex processing structures until the signals reach integration areas in sensory pathways. There are also connections in the opposite direction, which send feedback signals to the most primary areas, reinforcing the perception of stimulated sensation. Feedback signs from higher areas onto sensory areas reinforce expected stimuli, while inconsistent firings with the expected stimulus to other sensory areas are inhibited, for each sensory perception, in normal adults. In synesthesia, some of those inhibitory feedback mechanisms fail, allowing primary sensory areas to be activated by an unexpected stimulus from another sense. This hypothesis assumes that connections between the upper areas and sensory areas are not fully pruned, although they would normally be inhibited when they are not required to enhance the initial stimulus.^2^

The second thread relates to the architectural level of the model. Four hypotheses are discussed, which Hubbard^4^ terms local cross-activation, long-range disinhibited feedback, reentrant processing, and hyperbinding. The hypothesis of "local cross-activation" is linked to the anatomical assumption of failure of synaptic pruning. This model suggests similar activation to that observed in patients with phantom limbs, who maintain an activation of the perceptual field, given the persistence of the nerve. This model is more suitable to explain the grapheme-color-type synesthesia because the visual word form area (VWFA) is adjacent to the color-processing area (HV4), facilitating the persistence of a local connection. Research on monkey fetuses showed the existence of many connections between the lower temporal area (where the VWFA is located) and the V4 area, which are pruned in the prenatal phase and not present in adult monkeys.^2^ However, this model fails to explain other types of synesthesia, for example, when synesthesia is activated by a concept, including the names of days or months.

The long-range disinhibited feedback model^4^ is best applied to the physiological hypothesis of failure of the signal inhibition process, albeit without excluding the possibility of the anatomical hypothesis. This model suggests that synesthesia might result from disinhibited feedback in areas combining multisensory stimuli, including the temporo-parietal-occipital junction (TPO), where the angular gyrus is situated.

Reentrant processing is a hybrid model and an explanation which is more closely linked to the physiological hypothesis (inhibition failure) for types of synesthesia involving neighboring areas, wherein the grapheme-color synesthesia results from a signal reentry process. In addition to normal activity, which is directed from the primary visual area (V1) to the color-processing area (V4), then to the posterior inferior temporal region (PIT) and on to the anterior inferior temporal region (AIT), anomalous activity resends the signal in the AIT→ PIT →V4 direction, which would generate the synesthetic experience.^4^

Fourth is the hyperlink model.^30^ Under normal circumstances, the brain needs to gather information on form, color, and motion, among others, to construct a representation of the world, which relies on processing in the parietal lobe.^31^ Parietal hyper-activation of this process could be responsible for synesthetic experiences. A strong argument in favor of the hyperlink theory is the fact that most types of grapheme-color synesthesia are affected by the context, as in the example mentioned earlier of the character "l", which may be interpreted as a number or letter, depending on the context, with differing synesthetic perception.

Another model involving the connection of primary information for the construction of synesthetic perception is termed the model of the limbic bridge,^32^ which proposes that the synesthetic combination of sensory perceptions is conducted through the limbic system. According to this model, sensory information is emotionally evaluated by the limbic system and is connected through the limbic system when sensory data apparently has the same emotional rating. The concept of combining sensory perceptions through the limbic system was initially proposed by Cytowic,^13^ who observed limbic system activation in synesthetes using scintigraphy imaging methods. However, this model is not supported by the testimony of synesthetes, who generally report not perceiving any effect of synesthetic experiences on their affective states.^32^

The models above emphasize either an anatomical or a physiological hypothesis, although they are unable to exclude either. The physiological hypothesis also explains adventitious synesthesias, which occur because of a failure in the mechanism of feedback inhibition. Hubbard^4^ highlights that experiences triggered by psychedelic drugs are different from synesthesia because they are usually relatively more complex, pointing to different mechanisms. This notion is supported by Sinke et al.^32^ who, In their review article, compare the phenomenological characteristics of three types of synesthesias-congenital, acquired, and drug-induced-highlighting the much greater complexity and intensity of drug-induced synesthesias relative to congenital or acquired synesthesias. However, Cohen Kadosh et al.^33^ induced experiences similar to the grapheme-color synesthesia in non-synesthetes, through post-hypnotic suggestion. The association of colors with numbers was suggested to a group of participants during a hypnosis session. After waking, the participants were submitted to a test in which the numbers (written in black) were shown on a color screen. Participants missed significantly more digit identifications when the screen color coincided with that suggested for the number. The experiment reinforced the hypothesis of disinhibition between brain areas because there was insufficient time for the construction of new connections. Therefore, the researchers suggested that anatomical differences are not a prerequisite for synesthesia and raised the possibility that long-term disinhibitions (including in congenital or post-traumatic-acquired synesthesias) may lead to anatomical differences.

Eagleman^34^ raised the possibility that synesthesia is a mechanism encompassing, at least temporarily, a collection of various neural phenomena and that we are mistakenly trying to construe them all as one phenomenon.

Clearly, the study of synesthesia is in its infancy and there are still many controversies and loose ends, ranging from its definition and mechanisms to the perspectives and breadth of its study.

**Synesthesia and perception.** Research on the neurological condition of synesthesia has indicated that the phenomenon is not uniform, even for a single type of synesthesia. Rouw and Scholte^35^ differentiated grapheme-color synesthetes into projector and associator synesthetes, depending on whether the subjects experienced the synesthetic sensation (color display) in the external world in a specific area around them or internally in the "mind's eye". They also differentiated the degree of intensity of the sensation. The differences within the group of synesthetes were consistent with the level of structural connectivity found in each individual, and the higher the intensity of the synesthetic projection experience, the stronger the connectivity.

Several research studies have been conducted in the fields of multisensory processing and cross-sensory and cognitive modes in parallel with the study of synesthesia.^36,37^ These investigations have sought to understand how the brains constructs a perception of the world from various inputs, combining those considered congruent while segregating those evaluated as incongruent. Some terms have been used to designate the correspondence between senses, including synesthetic association or correspondence, or cross-modal correspondence or similarity. In general, the terms "synesthetic correspondence" and "synesthetic association" have only been used to describe correspondence between non-redundant sensory dimensions (for example, the correspondence between sound frequency and its association with brightness, in sight, without external stimulus). By contrast, other terms, such as "similarity" or "cross-modal correspondences", have a broader scope, including both synesthetic correspondences and correspondence between redundant stimuli (perceived through different sensory modalities), which refers to auditory and visual length of an event.

In one of these studies, Bien et al.^38^ examined the synesthetic correspondence between sound frequency and size. They started out from the hypothesis that a more general and subtle form of synesthesia would be the basis for the mechanisms underlying multisensory perception. The researchers resorted to the paradigm of ventriloquism to assess their hypothesis, wherein the illusion that a sound originates from an image is created in an audiovisual display of speech when the visual stimulus (lip movement) is separated from the spatial location of the sound source, although both are temporally and semantically congruent.^39^ In the experiment, they simultaneously presented bass or treble tones and large or small circles, and the subjects had to identify the location of the sound source. The task of locating the sound source was hampered upon synesthetic congruence between image and sound (bass tones-large images, treble tones-small images). The potential related to events (PRE) was measured while they performed the test. The subjects were submitted to a session of transcranial magnetic stimulation^40^ in the right intraparietal sulcus, a brain region relevant for both synesthetic perception and cross-modal processes,^38^ before a second test battery. Thus, the ventriloquism effect was temporarily interrupted, increasing the behavior of spatial location of the sound source. The origin of tone-shape synesthetic processing could be mapped by correlating the results from the behavioral test, transcranial magnetic stimulation, and event-related potentials with the involvement of the right intraparietal sulcus approximately 250 ms after stimulus onset. Their results provide evidence that synesthesia is located at one end of a spectrum of normal and adaptive perceptual processes, implying a close interrelationship between the different sensory systems.

This evidence suggests that a degree of structural connectivity exists, whereby the synesthetic sensation is no longer consciously experienced, although it still enables the person to perform the necessary associations to construct a perception of the world. Although some other research studies have failed to confirm the anatomical hypothesis of synaptic pruning, meaning the hypothesis of failed inhibition would prevail, the conjecture that synesthesia is a more exacerbated level of connectivity existing in all individuals remains.

Mirror-touch synesthesia apparently reinforces the notion that this perceptual condition is an exacerbated form of a mechanism present in ordinary perception. The most interesting aspect of this type of synesthesia stems from evidence of the existence of a system of neurons in humans, termed the mirror-neuron system, that are activated not only when performing a task but also when observing another person performing the task.^41^ This form of synesthesia most likely occurs through the hyper-activation of this normal system.

Fitzgibbon et al.^42^ addressed a special case of mirror-touch synesthesia, pain synesthesia, comparing it with pain empathy in a review article. Evidence that pain empathy could be mediated by a system of mirror neurons emerged with the discovery of neurons in the anterior cingulate cortex that fire, in normal individuals, both in response to the sensation of pain and upon observing another person in a painful situation, according to the authors. The authors found that the activity of these neurons is exacerbated, surpassing a threshold that causes the experience to be consciously perceived, both in pain synesthetes and in mirror-touch synesthetes. There is a key difference between mirror-touch synesthesia and pain synesthesia: while the former is apparently innate, cases of pain synesthesia are only found as a result of trauma, especially after amputation. These cases reinforce a physiological explanation of exacerbation of neural activity as an emotional response, at least for this type of synesthesia.

The study of synesthesia has contributed greatly to our understanding of perceptual processes and creative capacity. Metaphor involves the connection of high-level concepts, most likely embedded in different brain regions, just as synesthesia involves the connection of different sensory entities.^43^ It is hypothesized that "the angular gyrus, which is disproportionately larger in humans than in apes and monkeys, evolved originally for cross-modal associations but then became co-opted for other, more abstract functions such as metaphors."^43^

Ward et al.^44^ found, in a study conducted with 82 individuals with various types of synesthesia, that synesthetes have a significant tendency to devote more time to artistic activities related to their type of synesthesia and suggest that synesthetes may have better access to specific associations.

Day^21^ realized that the construction of metaphors in human language is not random and follows specific patterns, when comparing the neurological condition of synesthesia with synesthetic metaphors. The author compared the prevalence rates of synesthesias surveyed by Cytowic^45^ with synesthetic metaphors in the English and German languages, concluding that sound is the primary sensation that most arouses secondary synesthetic sensations and is also the primary sensation on which we construct the highest number of metaphors. Unlike the neurological condition of synesthesia, which is idiosyncratic, at least regarding the grapheme-color synesthesia, the supposed latent synesthesia is apparently shared and has a key role in the conformation of perception.

Parise and Spence^46^ examined the role of synesthetic correspondences in the integration of pairs of spatially or temporally conflicting auditory and visual stimuli. Stimuli tend to unite when they both have a high degree of combination, masking the sensory origin of each stimulus. Their results showed that small figures have a high degree of combination with treble, while larger figures combine more with bass.

**Figure 3:**
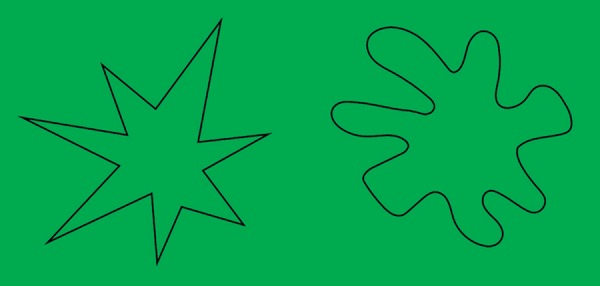
Kiki and Booba. Association between the sound of words and the shape and color of the figures.^43^

An experiment that highlights the sharing of synesthetic perceptions was conceived by the Gestaltist Wolfgang Köhler,^43^ in which subjects are asked to associate the names Kiki and Booba to the figures (Figure 3)

Approximately 95 to 98% of people choose Kiki for the orange, angular shape and Booba for the round, violet shape. Inspired by the paradigm of the "Kiki and Booba" test, we created an experiment to test the two-way association between four musical excerpts of approximately 50 seconds with four synesthetic adjectives (Table 1).^22^ There are 24 possible combinations between songs and adjectives. This means that the distribution of responses would follow a random pattern in which each combination comprises 4.17% of responses if the songs aroused no synesthesia or if they were totally subjective (non-shared); two to three of the 64 responses collected would be assigned to each combination.

**Table 1:** “Kiki or Booba”-type test for music.^22^

Luciano Berio, Folk Songs - I wonder as I Wander	Sweet
Luciano Berio, Chamber Music – Monotone	Static
Ravel, Le tombeau de Couperin - I. Prélude	Light
Penderecki, Seven Gates of Jerusalem, “Symphony No. 7” - V. Lauda Jerusalem	Heavy

The results showed 58 (90.6%) similar responses, which attributed the adjectives to the songs as listed in Table 1. This convergence of results is comparable to the aforementioned "Kiki and Booba" experiment, bearing in mind that the random pattern is 50% in the "Kiki and Booba" test. There is also a factor that makes the convergence of results from the experiment even more remarkable: both figures may be simultaneously shown in the visual "Kiki and Booba" experiment, whereas participants must resort to their memory to compare the songs in the experiment with musical excerpts.

## Conclusion.

The definition of synesthesia, its basic characteristics and limits are still far from being clearly established, although its study may generate significant advancements to human knowledge.

There is a fairly thin line between weak synesthetic conditions and cross-modal sensory and cognitive perception, which is apparently a key area in the construction of abstract associations between different perceptual fields and is crucial in musical perception. Musical meaning might be largely constructed by synesthetic processes whereby sensory associations from sound activate memories, images, and emotions. Music is most likely the ideal field of knowledge for the study of synesthesia because it is the human activity where this phenomenon is most explicit.

We hypothesized that synesthesia is a key mechanism in music perception, affecting the induction of emotions, even when the synesthetic level is not explicit (conscious). Future progress in the study of synesthesia may bear fruit for music composition, analysis, and especially pedagogy and therapy.
